# Long-Term Studies Reveal Differential Responses to Climate Change for Trees Under Soil- or Herbivore-Related Stress

**DOI:** 10.3389/fpls.2019.00132

**Published:** 2019-02-18

**Authors:** Amy V. Whipple, Neil S. Cobb, Catherine A. Gehring, Susan Mopper, Lluvia Flores-Rentería, Thomas G. Whitham

**Affiliations:** ^1^Department of Biological Sciences, Merriam-Powell Center for Environmental Research, Northern Arizona University, Flagstaff, AZ, United States; ^2^Department of Biology, University of Louisiana at Lafayette, Lafayette, LA, United States; ^3^Department of Biology, San Diego State University, San Diego, CA, United States

**Keywords:** climate change, drought, growth, herbivory, long-term, reproduction, tree

## Abstract

Worldwide, trees are confronting increased temperature and aridity, exacerbating susceptibility to herbivory. Long-term studies comparing patterns of plant performance through drought can help identify variation among and within populations in vulnerability to climate change and herbivory. We use long-term monitoring data to examine our overarching hypothesis that the negative impacts of poor soil and herbivore susceptibility would be compounded by severe drought. We studied pinyon pine, *Pinus edulis*, a widespread southwestern tree species that has suffered extensive climate-change related mortality. We analyzed data on mortality, growth, male reproduction, and herbivory collected for 14–32 years in three areas with distinct soil-types. We used standardized precipitation-evapotranspiration index (SPEI) as a climate proxy that summarizes the impacts of drought due to precipitation and temperature variation on semi-arid forests. Several key findings emerged: (1) Plant performance measurements did not support our hypothesis that trees growing in stressful, coarse-textured soils would suffer more than trees growing in finer-textured soils. Stem growth at the area with coarse, young cinder soils (area one) responded only weakly to drought, while stem growth on more developed soils with sedimentary (area two) and volcanic (area three) substrates, was strongly negatively affected by drought. Male reproduction declined less with drought at area one and more at areas two and three. Overall mortality was 30% on coarse cinder soils (area one) and averaged 55% on finer soil types (areas two and three). (2) Although moth herbivore susceptible trees were hypothesized to suffer more with drought than moth resistant trees, the opposite occurred. Annual stem growth was negatively affected by drought for moth resistant trees, but much less strongly for moth susceptible trees. (3) In contrast to our hypothesis, moths declined with drought. Overall, chronically water-stressed and herbivore-susceptible trees had smaller declines in performance relative to less-stressed trees during drought years. These long-term findings support the idea that stressed trees might be more resistant to drought since they may have adapted or acclimated to resist drought-related mortality.

## Introduction

Heightened drought severity and warming temperatures due to climate change have contributed to world-wide increases in tree mortality (Allen et al., 2010; McDowell et al., 2018). Physiological drivers of mortality include hydraulic failure and carbon starvation (McDowell et al., 2011; Adams et al., 2017) with drought leading to mismatches between the water demand of aboveground tree biomass and water availability in the soil (Jump et al., 2017). Herbivorous insects and fungal pathogens can exacerbate drought impacts on trees (Allen et al., 2010; Anderegg et al., 2015a). Drought affected forests consist of areas that differ significantly in mortality (Mueller et al., 2005a; Gitlin et al., 2006; Linares et al., 2011; Olano et al., 2015), and trees that live and die following severe drought often grow side by side (Ogle et al., 2000; Sthultz et al., 2009a). Given that the incidence of severe drought is projected to increase (Seager et al., 2007; Garfin et al., 2013; IPCC, 2014; United States Global Change Research Program [USGCRP], 2017), and that tree mortality leaves a legacy that alters carbon cycling (Anderegg et al., 2015b), understory community composition (Kane et al., 2011), and community interactions (Gilman et al., 2010), it is critical to understand the factors that contribute to variation in tree mortality. It is equally important to understand how surviving trees and their pests respond following drought to better predict the future of forested ecosystems.

The impacts of drought on trees may differ across the landscape due to differences among sites in soil texture and water holding capacity. Mortality of trees was associated with low water holding capacity due to a combination of soil depth and texture in the semi-arid southwestern United States (Peterman et al., 2013). Stands with hotter, drier climates and low soil available water capacity showed limited regeneration following drought-related tree mortality (Redmond and Barger, 2013; Redmond et al., 2015). However, soil texture did not influence tree mortality following severe drought in the Amazon Basin (Williamson et al., 2000). Also, the effects of soil properties may vary temporally as the precipitation regime changes. The inverse texture hypothesis (Noy-Meir, 1973) proposes that plants growing in coarse-textured soil experience less water stress than plants growing in fine-textured soil and Sala et al. (1989) suggested this soil texture effect differed with annual precipitation. Longer-term data on tree performance at sites that differ in soil properties and that include drought and non-drought years could help reconcile conflicting patterns.

Intraspecific variation in drought tolerance can be significant in trees (Goodrich et al., 2016; Trujillo-Moya et al., 2018) and associated with differences in mortality during extreme drought (Sthultz et al., 2009a; Gehring et al., 2017) and growth recovery following drought (George et al., 2017). Common garden studies conducted across drought periods in *Larix decidua* (George et al., 2017) and *Pinus sylvestris* (Taeger et al., 2013) revealed significant differences among provenances in a tree’s capacity to both withstand drought and to reach pre-drought growth levels after drought. Similarly, intraspecific variation in Norway spruce (*Picea abies*) explained up to 44% of the phenotypic variation in drought response (Trujillo-Moya et al., 2018). Many studies demonstrating the importance of intraspecific genetic variation in drought response compare individuals of widely distributed plant species that occupy markedly different environments. It is less clear how intraspecific genetic differences contribute to the variable levels of mortality observed among trees occupying the same or similar sites.

Differential herbivory also can contribute to differences in tree performance under drought conditions. Drought interacts with insect herbivory by altering plant defenses, influencing water and nutrient content of plant tissues, and altering chemical cues used by insects to identify hosts (Kolb et al., 2016a). While these interactions can lead to herbivore outbreaks that increase tree mortality (Ayres and Lombardero, 2000; Breshears et al., 2005; Raffa et al., 2008; Allen et al., 2010), drought can also reduce resource quality for herbivores, resulting in herbivore population declines (Kolb et al., 2016a). Insect herbivores also can be affected directly by the high temperatures often associated with summer drought. For example, warm temperatures combine with drought-stressed susceptible hosts increase bark beetle populations to epidemic levels (Raffa et al., 2008). Studies that examine both tree and herbivore performance over time can help us understand the contribution of herbivory to declines in tree performance with drought.

While differences in soil properties, intraspecific trait variation, and insect herbivory all influence susceptibility to, and recovery of trees from drought, these factors are rarely studied simultaneously. Because trees are long-lived and integrate climatic variation across multiple years, it is especially important to evaluate these complex interactions over the long term as climate changes (Linares et al., 2011; Anderegg et al., 2015b; George et al., 2017). Long-term studies are well suited for exploration of changes in relationships over time. One example is the change in responses of C_3_ plants, which initially responded positively to elevated atmospheric CO_2_, versus C_4_ grasses which responded positively at 20 years (Reich et al., 2018). Furthermore, by comparing precipitation manipulation experiments to studies of productivity responses to annual precipitation variation across space versus across time, Estiarte et al. (2016) found that studies across time were more accurate predictors of future responses. Thus long-term studies across time and space that encompass strong variation in precipitation are critical to understanding responses to drought.

In this study we use a model tree species, pinyon pine (*P. edulis*) to examine how soil type, intraspecific variation in drought tolerance and herbivore susceptibility interact to influence tree performance before and during long-term drought. Warming temperatures combined with extreme drought and herbivory resulted in significant *P. edulis* mortality across 12,000 km^2^ of the southwestern United States in 2002–2003 (Breshears et al., 2005). Several studies have examined the physiological basis and environmental drivers (Adams et al., 2009, 2015, 2017; Peterman et al., 2013; Grossiord et al., 2017; Redmond et al., 2017; Guérin et al., 2018) of this large-scale mortality. A synthesis of this research indicated significant differences among studies in the importance of tree density, site elevation and soil type to mortality (Meddens et al., 2015). Also, while the contribution of bark beetle herbivory to tree mortality has been demonstrated in *P. edulis* (Santos and Whitham, 2010; Gaylord et al., 2013; Anderegg et al., 2015a), other insect herbivores affect *P. edulis* performance and may influence its future distribution. Larvae of the pinyon stem-boring moth (*Dioryctria albovittella*) feed preferentially on the terminal shoots and female cones of mature trees. In studies conducted before long-term drought, chronic herbivory by this moth reduced trunk growth and female cone production, and altered *P. edulis* architecture to a shrub-like form (Whitham and Mopper, 1985). Despite their poor growth prior to drought, moth susceptible trees had three-fold higher survival during drought than moth resistant trees (Sthultz et al., 2009a), a trait also observed in their offspring (Sthultz et al., 2009b; Gehring et al., 2017).

In their synthesis of the drivers of *P. edulis* mortality, Meddens et al. (2015) developed a conceptual framework describing how soil, climate, abiotic agents, and tree attributes relate to *P. edulis* mortality via the physiological mechanisms that contribute to mortality. We follow that framework in the development of our hypotheses. Our overarching hypothesis is similar to that of Meddens et al. (2015) in that we propose that the negative impacts on *P. edulis* of growing in poor soil (an abiotic stressor), and experiencing chronic herbivory (a biotic stressor), would be compounded by severe drought. The hypotheses we describe were developed when our monitoring program began in the 1980’s and 1990’s, before some contradictory observations had been made (Sthultz et al., 2009a; Meddens et al., 2015; Gehring et al., 2017). We use long-term data on mortality, stem growth, male reproduction, and moth herbivory of *P. edulis* trees at areas in northern Arizona to test the following hypotheses: (H1) trees growing in shallow, nutrient-poor, coarse-textured soil with low water holding capacity will suffer greater declines in performance than trees in areas with deeper, higher nutrient, fine-textured soil with higher water holding capacity under drought conditions; (H2) trees experiencing chronic moth herbivory would suffer compounded declines in performance due to drought compared to herbivore resistant trees. We also examine long-term trends in levels of moth herbivory to test the hypothesis that moth herbivory would increase as trees experienced greater drought (H3). We expected moth herbivory to increase with drought stress because *D. albovittella* is more abundant in stressful sites with shallow, coarse-textured, nutrient-poor soils (Gehring and Whitham, 1994; Cobb et al., 1997).

These hypotheses are evaluated in context of the slope of the relationship of tree and herbivory measures regressed on the standardized precipitation-evapotranspiration index (SPEI) (Vicente-Serrano et al., 2010; Beguería et al., 2014). SPEI integrates precipitation, temperature, and evapotranspiration and is a proxy for drought stress impacts on plants that can be tailored to the timing of the system (Huang et al., 2015; George et al., 2017). SPEI is a relative measure of drought stress within a particular location with a mean of zero over time, where relatively warm and dry conditions are indicated by negative values. SPEI does not capture differences among our areas in water availability due to soil differences, rather it allows us to track the effects of drought through time within an area. Based on previous results in southwestern United States tree species, we chose to use SPEI over the year preceding a performance measure (Williams et al., 2013; Huang et al., 2015). Significant positive relationships of performance measures with SPEI indicate decreased performance under drought stress, while no significant relationship indicates that performance remains relatively constant with increasing drought stress. Our hypotheses would be supported by slopes of regression of tree performance measures with SPEI that are greater for the coarse soil (H1) and herbivore susceptible (H2) groups. For moth herbivory, we predict a negative slope with SPEI, which would indicate that the number of moth killed stems increases with drought stress (H3). Tests of these hypotheses are important to identify the long-term responses of individual trees and an associated herbivore to drought stress. Our findings may help land managers mitigate the trajectory of *P. edulis*, which is projected to be extirpated from Arizona this century due to climate change (Rehfeldt et al., 2006).

## Materials and Methods

### Characteristics and Soils of the Three Areas

All three study areas are woodlands dominated by *P. edulis* and *Juniperus monosperma*. Study area one is near Sunset Crater National Monument on the cinder field associated with an ∼1000 years old volcanic eruption (Table 1). This area has coarse-textured soils with low water holding capacity and is represented by six sub-sites while study areas two and three have finer-textured soils with greater water holding capacity and are represented by three sub-sites each (Gehring and Whitham, 1994; Miller et al., 1995; Selmants and Hart, 2008, Table 1). Cinder soils of area one have lower nutrient levels of nitrogen, phosphorus, potassium, magnesium, calcium, sodium, copper, and manganese compared to soils developed on limestone and sandstone substrates (area two) (Cobb et al., 1997; Swaty et al., 1998). Other studies have also shown the young, cinder soils to be lower in phosphate, soil moisture, NO_3_ mineralization, and NH_4_ mineralization (Gehring and Whitham, 1994, 1995). Additional studies comparing area one to area three, which is a much older volcanic-substrate soil, also found area one to be lower in clay, nitrogen, carbon, and soil water (Selmants and Hart, 2008; Selmants and Hart, 2010; Looney et al., 2012). Area three is lower than area one in plant available forms of phosphorus, as would be predicted for an older, versus younger, volcanic soil (Selmants and Hart, 2010). Because of generally lower nutrient status and low water holding capacity, trees growing in the cinder soils of area one experience more stressful abiotic conditions. The trees at the young cinder soil sites c,d (see Table 1) of area one exhibit higher incidence of susceptibility to the stem-boring moth (*D. albovittella*) and studies of moth susceptible and resistant trees in these areas began in the 1980’s (see references under H2 and H3 sections below) hence hypotheses H2 and H3 will be addressed with trees from area one only. Annual stem growth measures are available starting with year 1986, strobili counts starting in year 1995, and moth killed stems (H3 only) starting in year 1982.

**Table 1:** This design table for the cross soil-type area comparisons in H1 shows the locations and number of trees sampled (N) for each the sampling area and the sub-sites within them.

Area	Sub-site	Latitude	Longitude	*N*	Soil characteristics (from Miller et al., 1995)
1	a	-111.39	35.336	63	Deep, extremely cindery sandy loam, excessively drained
1	b	-111.41	35.39	60	Deep, extremely cindery, coarse sand, excessively drained
1^∗^	c,d	-111.43	35.39	122	Deep, extremely cindery, coarse sand, excessively drained
1	e	-111.46	35.418	64	Deep, extremely cindery, coarse sand, excessively drained
1	f	-111.48	35.44	64	Deep, extremely cindery, coarse sand, excessively drained
2	a	-111.41	35.15	64	Shallow, fine sandy loam
2	b	-111.42	35.18	65	Shallow, fine sandy loam
2	c	-111.4	35.13	62	Deep, fine sandy loam
3	a	-111.84	35.52	65	Moderately deep, very cobbly, clay loam
3	b	-111.84	35.54	64	Moderately deep, very cobbly, clay loam
3	C	-111.86	35.55	64	Deep, very cindery loam


*Sub-sites consist of a set of sampled trees that are contiguous and distinct from other sub-sites within an area. Areas one and three are on volcanic substrates and area two is sedimentary. The asterisk denotes sub-sites that where merged for analyses because they were spatially contiguous. Trees for H2 and H3 are in area one sub-site “c,d”.*

### Trait Measures

When trees were selected for the monitoring program, their basal trunk diameters and heights were measured. Stem growth (length added in a year) was measured after the conclusion of growth in late summer or fall on eight haphazardly selected stems from around the entire tree. The stem growth record was extended back in time by using bud scars as indicators of growth in years prior to the first measurement date (Ewers and Schmid, 1981). When possible, this method was also used to fill in years that measures were missed over the course of the 14–32 years of monitoring. Male strobili clusters (pollen cones) were counted in the spring; each tree was counted twice and the two counts were averaged. Moth killed stems show a characteristic browning and wilting that was used for visual identification (Whitham and Mopper, 1985; Gehring and Whitham, 1994). Moth killed stems were counted in the late summer and early fall after the moth larvae had completed feeding. Ladders were used to view the tree canopy as needed. Moth killed stems were counted twice on each tree and the two counts were averaged. Tree mortality was monitored beginning in the extreme drought year of 2002 and was rare prior to 2002.

### Hypothesis 1: Tree Performance Across Three Areas/Soil Types

Thirty-two medium and 32 small trees in each of the replicate sites (Table 1) within an area/soil-type were selected haphazardly by finding interspersed trees meeting height and basal trunk diameter requirements for small and medium categories of trees. Most trees were selected in 1995, but a few more trees were added through 1999 to account for tree mortality if there were suitably sized trees available within the sub-site. Small trees had mean basal trunk diameter (±1 SE) of 6.42 cm (±0.08) and medium trees had basal trunk diameter of 15.25 cm (±0.17) at the start of the study. Table 1 shows the locations of replicate sites within areas that make up the nested geographic design. This design increased the generality of the comparison across areas with contrasting soils type by increasing spatial representation. Total sample sizes were: area one = 373, area two = 191, area three = 193 (Table 1). Stem growth was measured for years 1986–2007. We counted male strobili clusters on each tree from year 1995–2008.

### Hypothesis 2: Relative Performance of Moth Resistant and Susceptible Trees Within Area One

At the main cinder soil study area, 25 moth resistant and 25 susceptible trees were chosen for long-term monitoring and have been reported on previously (Brown et al., 2001; Cobb et al., 2002; Gehring et al., 2017). The trees are closely matched in age and basal trunk diameter, and the height difference reflects the tree versus shrub architecture that results from moth herbivory (Brown et al., 2001; Cobb et al., 2002). Linear models found significant differences among tree groups only for height and not for basal trunk diameter or age. Moth resistant trees had basal trunk diameters of mean = 20.3 cm (*SE* = 0.73) and heights of mean = 3.8m (*SE* = 0.13) at the start of monitoring. Moth susceptible trees had basal trunk diameters of mean = 18.9 cm (*SE* = 0.62) and heights of mean = 2.2 m (*SE* = 0.087) at the start of monitoring. Stem growth and strobili production were monitored annually as described above, except during the extreme drought year of 2002 when new growth was not produced on most trees.

### Hypothesis 3: Moth Relative Abundance Monitoring Within Area One

The number of moth killed stems was monitored from 1981 to 2009 on large moth susceptible (*N* = 20) and resistant trees (*N* = 19) in the main cinder soil study area. These trees have been reported on previously (Whitham and Mopper, 1985; Gehring and Whitham, 1991; Mopper et al., 1991; Brown et al., 2001). Resistant trees had basal trunk diameters of mean = 44.5 cm (*SE* = 2.2) and heights of mean = 6.55 m (*SE* = 0.21) at the start of monitoring. Susceptible trees had basal trunk diameters of mean = 42.4 cm (*SE* = 1.9) and heights of mean = 3.8 m (*SE* = 0.13) at the start of monitoring. The analysis of moth herbivory will focus on these large trees because of the power available in the longer record that includes more non-drought years.

### Climate Proxy and Analyses

We chose 12-month periods for annual calculations of the Standardized Precipitation Evapotranspiration Index, SPEI, as our climate proxy based on: (1) the known sensitivities of pinyon pine to precipitation, summer heat, and vapor pressure deficit (Adams et al., 2009, 2015, 2017; Grossiord et al., 2017; Guérin et al., 2018), (2) the success of SPEI and similar measures for assessing drought effects on trees in southwestern forests (Williams et al., 2013; Anderegg et al., 2015b; Huang et al., 2015; Redmond et al., 2017), and (3) the annual time increment inherent in our data. This measure allowed us to assess the basic patterns of growth, male reproduction, and moth herbivory as correlated with the important climate drivers incorporated into SPEI (precipitation and estimated evapotranspiration as influenced by temperature), while acknowledging that climate data sparseness and lack of inclusion of soil information means this metric will not fully represent drought stress variation in time and space for our individual study sites (Kolb, 2015). SPEI, was calculated and downloaded from the Desert Research Institute website for each of the 11 sites at which trees were monitored (Vicente-Serrano et al., 2010; Abatzoglou et al., 2017). We used the SPEI calculated based on the 12 months prior to September of the measurement year for stem growth and moth killed stem relationships. For the male strobili count measures, we use SPEI for the 12 months prior to May of the measurement year since strobili appear in May. In some cases, sub-sites within areas 1–3 were close enough that the values for interpolated SPEI were identical.

For stem growth area comparisons, we used the lme procedure in the NLME package in R to conduct autoregressive, moving average, mixed-model analysis that allows us to account for time lags (Crawley, 2012; Pinheiro et al., 2018). Tree was always included as a random factor in time (repeated measure) in these analyses. The repeated measures analyses allowed us to accommodate individuals that were added after the initial start of sampling and individuals that died after the start of data collection. We used the time series functionality for lme to test for autoregressive and moving average effects across time lags of 1 and 2 years. The Akaike Information Criterion (AIC) was used for selecting among these models with different correlation structures in time (Burnham and Anderson, 2004). We compared autoregressive (AR) and moving average (MA) effects for *t* = 0, 1, or 2 years in an ARMA (*p*,*q*) model where *p* = the AR lag and *q* = the MA window, because previous work suggested the likelihood of lag effects (Anderegg et al., 2015b).

Our stem growth and strobili count models for the comparison of patterns with drought (SPEI) across areas with contrasting soil types (H1) included an area fixed effect for the three areas with distinct soil types, SPEI and its interaction with areas, height and basal trunk diameter as well as random effects for sites with areas and for individual trees across years. We included the initial size measures as covariates instead of small and medium size categories to increase the power of the analysis. The results were similar when the size groups were analyzed separately. The correlation coefficient for the basal trunk diameter and height is 0.64, which is low enough to justify including both measures of size. For male strobili counts, we used a Poisson link function in a mixed-model, repeated measures analysis in glmmTMB (Brooks et al., 2017). Mortality for the area comparisons was analyzed in a binomial model of cumulative mortality between 2002 and 2007 since this data gave us enough information content for a meaningful analysis.

The data sets for H2 and H3 were not large enough to meaningfully test for autocorrelation in time. These monitoring data sets include trees at just one site. Because these trees are more uniform in size than the trees in the area comparisons, and the sample size was smaller, we did not include size-related covariates in the analyses for H2 and H3. For stem growth, we again used the lme procedure and for strobili and moth-killed stem counts we used glmmTMB with a Poisson link function.

## Results

### Model Selection and Multi-Year Effects

The results of model selection for choosing the time period and type of lags included in the final model of stem growth by area are in Table 2. In addition to correlations in time, our stem growth models for the comparison of patterns with drought across areas with contrasting soil types included an area fixed effect for the three areas with distinct soil types, SPEI and its interaction with area, height and basal trunk diameter and random effects for sub-sites within area and for individual trees across years. We choose the ARMA (2,1) model with a moving average window, MA = 2 and autoregressive lag, AR = 1. The model choice was based on the criteria of having an AIC more than two lower than any other model (the difference in AIC was 3.3), as suggested by Burnham and Anderson (2004). The next best model was MA = 1, AR = 2 and gave similar significances and parameter estimates for the fixed effects in the model. Based on these results, there are time lag effects for growth in this system, but their exact form is less certain since some models are similar in AIC. These lag effects indicate that multiple years of drought had greater effects together than when drought years were isolated. Variation in the ARMA time lags included in the model does not alter the basic patterns observed in significance of the fixed factors or covariates, and only makes slight modifications to the magnitude of the coefficients for these parameters.

**Table 2:** Model selection table used to choose the form of correlations in time for the analysis of stem growth and drought across areas/soil types.

Y	Significant fixed factors and covariates	MA, *p*=	AR, *q*=	AIC
Stem growth	Area, SPEI, Area^∗^SPEI, height, btd	0	0	90253.4
Stem growth	Area, SPEI, Area^∗^SPEI, height, btd	0	1	88466.9
Stem growth	Area, SPEI, Area^∗^SPEI, height, btd	0	2	87363
Stem growth	Area, SPEI, Area^∗^SPEI, height, btd	1	0	87164.7
Stem growth	NA	1	1	Singular
Stem growth	SPEI, Area^∗^SPEI, height, btd	1	2	86533.8
Stem growth	Area, SPEI, Area^∗^SPEI, height, btd	2	0	86693.3
Stem growth	SPEI, Area^∗^SPEI, height, btd	2	1	86530.1
Stem growth	SPEI, Area^∗^SPEI, height, btd	2	2	86528.6


*ARMA analysis in the nmle package in R allowed us to test MA, moving average windows; AR, autoregressive time lags of 0, 1, and 2 years each. The fixed factors and covariates included in each model are the same and sub-site is nested within area for all models. Model selection was based on having an AIC at least “2.0” less than the next best model. The model chosen is highlighted in gray and has a MA = 2 and AR = 1. Model output details for the selected model is in Table 3A. btd, basal trunk diameter.*

### Hypothesis 1: Performance of Trees in Areas With Different Soil-Types

**FIGURE 1:**
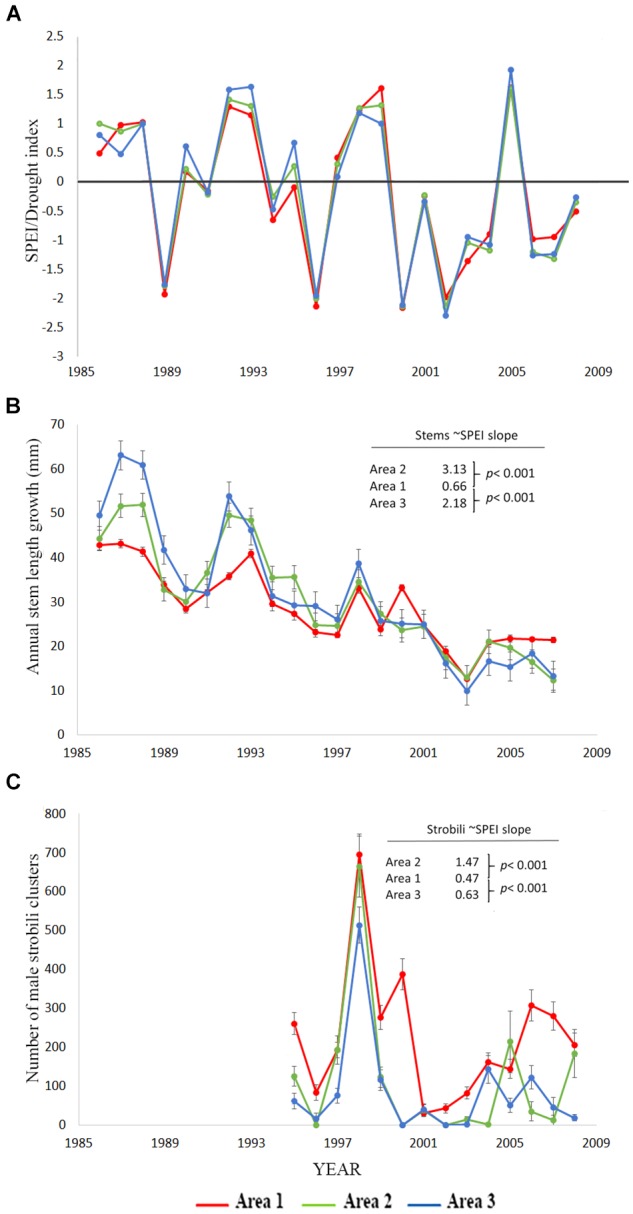
Data for the cross soil-type/area comparisons testing H1. **(A)** Standardized precipitation-evapotranspiration index, SPEI from the DRI website for the 12 months preceding the completion of annual stem growth (Abatzoglou et al., 2017) for which negative numbers indicate greater drought stress. SPEI for the strobili count analysis is different from the one used for stem measures as it is based on the 12 months prior to pollen production in May. Area one is the stressful, coarse soil site. **(B)** Annual stem length growth mean ± SE for the three areas. The coefficients provided are the slopes of the stem growth relationship to SPEI from the mixed model ARMA analysis in Table 3A and the *p*-values are tests of significant difference between the area one slope versus areas two and three. **(C)** Number of male strobili clusters mean ± SE for the three areas. The coefficients provided are the slopes of the male strobili count relationship to SPEI from the repeated measures Poisson link function analysis in Table 3B and the *p*-values are tests of significant difference between the area one slope versus areas two and three.

We hypothesized that trees growing at areas determined to be abiotically stressful due to coarse soil texture and low water holding capability before drought would suffer the greatest declines in performance as measured by stem growth, male strobili production, and mortality, but instead observed the opposite patterns. Trees in the coarse soil area (area one), did not suffer greater loss of performance during drought than the other areas (Figure 1 and Table 3). While Figure 1 shows the actual data and a rough correspondence between SPEI and performance measures, it does not show the full extent of the relationship (slope) between drought and stem growth because it does not account for the time series aspects of the data that are incorporated in the model. For area one, there was a significant, but small, positive slope for the relationship between stem growth and SPEI (slope = 0.66, SE = 0.10, slope different from 0: *p* < 0.001) so stem growth decreased slightly with increased drought. The other two areas had significantly greater slopes of the relationship between stem growth and SPEI when compared to area one (area two slope = 3.13, *p* < 0.001 and area three slope = 2.18, *p* < 0.001, Table 3A). These regression results indicate a lower responsiveness of growth to drought in area one (Figure 1 and Table 3A). Taken together, these results show that trees in area one respond less to drought than expected.

**Table 3:** Statistical analysis for comparisons of areas response to drought (H1).

(A) Annual stem length growth: ARMA model.					
	**Value**	**Std. error**	**DF**	***t*-value**	***p*-value**

Intercept (=area 1)	14.49	1.48	11516	9.786	0.0000
Area 2	0.73	1.64	8	0.443	0.6693
Area 3	1.73	1.64	8	1.054	0.3229
SPEI (=area 1 slope)	0.66	0.10	11516	6.954	0.0000
Basal trunk diameter	0.35	0.09	728	3.955	0.0000
Height	5.99	0.84	728	7.134	0.0001
Area 2^∗^SPEI	2.47	0.17	11516	14.148	0.0000
Area 3^∗^SPEI	1.52	0.16	11516	9.223	0.0000

**(B) Number of male strobili clusters: poisson link function.**					

	**Estimate**	**Std. error**	***z*-value**	**Pr(>|*z*| )**

Intercept (=area 1)	-3.33	0.873	-3.8	0.0001	
Area 2	-1.14	0.641	-1.8	0.0752	
Area 3	-0.75	0.603	-1.2	0.2163	
SPEI (=area 1 slope)	0.47	0.001	467.2	0.0000	
Basal trunk diameter	0.38	0.022	17.2	0.0000	
Height	1.45	0.200	7.3	0.0000	
Area 2^∗^SPEI	1.00	0.004	269.0	0.0000	
Area 3^∗^SPEI	0.16	0.003	58.1	0.0000	


***(A)** Stem length growth was modeled in an autoregressive-moving average framework with fixed factors and covariates of area/soil type, drought index (SPEI), the interaction of SPEI and area, initial tree basal trunk diameter and initial tree height. See Table 2 for model selection resulting in a moving average window = 2 years and an autoregressive parameter = 1 year for the model below. ***(B)*** Number of male strobili clusters were modeled repeated measures mixed-model with a Poisson link function with fixed factors and covariates of area/soil type, drought index (SPEI), the interaction of SPEI and area, initial tree basal trunk diameter and initial tree height. Random factors in both analyses included sub-sites within areas and individual trees across years. The comparisons of interest are primarily the slopes of the SPEI covariate and how it varies across areas. The intercept and slope for SPEI represent area one and coefficients for areas two and three and their interaction with SPEI represent the degree to which they differ from area one (testing H1).*

Data on male strobili counts also contradicted our hypothesis for the performance of trees growing in the coarse soils of area one relative to areas two and three. Male strobili counts were positively correlated with SPEI (Figure 1 and Table 3B) in all areas, indicating that strobili production was negatively affected by drought stress. The SPEI in Figure 1 is shifted a few months from the one used for the modeling of male strobili counts because strobili are produced months before growth is completed for the year. Although strobili production declined in all three areas, the slope of the relationship between SPEI and strobili production was low in area one (slope = 0.47, *p* < 0.001, Poisson link function) relative to areas two and three, which had greater decreases in strobili with drought (slope area two = 1.47, *p* < 0.001 for the contrast with area one and slope area three = 0.63; *p* < 0.001 for the contrast with area one, Table 3B).

**FIGURE 2:**
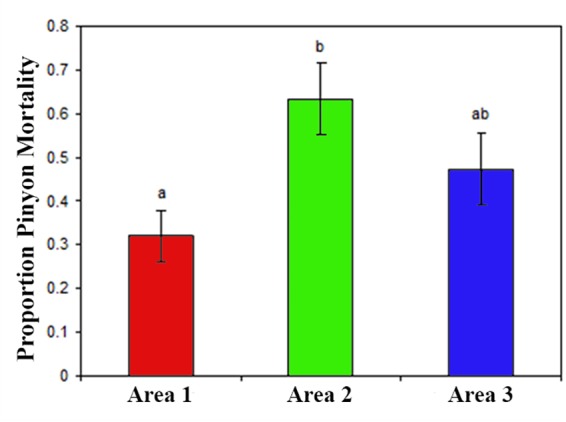
For the cross soil-type/area comparison (H1) these data are the cumulative mortality from 2002 to 2007 across the three areas. Significant differences as indicated by different letters above the bars come from the binomial mixed model in Table 4.

Also in contrast to hypothesis 1, mortality was not greater for area one (cinder soils) in comparison to the other areas (Figure 2 and Table 4), but instead was either significantly lower or similar. The binomial model of mortality shows that area one had a significantly lower proportion dead (0.30) than area two (0.63, *p* = 0.00186). We were not able to distinguish the proportion dead at area three (0.48) from area one (*p* = 0.14036). Lack of statistical power for this test limits interpretation for area three relative to area one, but it is unlikely that the true mortality is higher for area one.

**Table 4:** Mortality across area/soil type comparisons was tested (H1) with a mixed, binomial model of cumulative mortality from 2002 to 2007, a time of extreme drought with ongoing mortality.

	Estimate	Std. error	*z*-value	Pr(>|*z*| )
Intercept (=area 1)	-0.66	0.38	-1.714	0.0866
Area 2 difference from intercept	1.50	0.49	3.112	0.0019
Area 3 differences from intercept	0.71	0.48	-1.474	0.1404
Basal trunk diameter	-0.04	0.02	-1.764	0.0778
Height	0.15	0.20	0.784	0.4333


*Sub-sites were random factors nested within areas. Area one is represented by the intercept and area two and three coefficients represent differences from that intercept. Area two has significantly higher mortality than area one (Figure 2).*

### Hypothesis 2: Performance of Moth Susceptible Versus Moth Resistant Trees Within Area One

**FIGURE 3:**
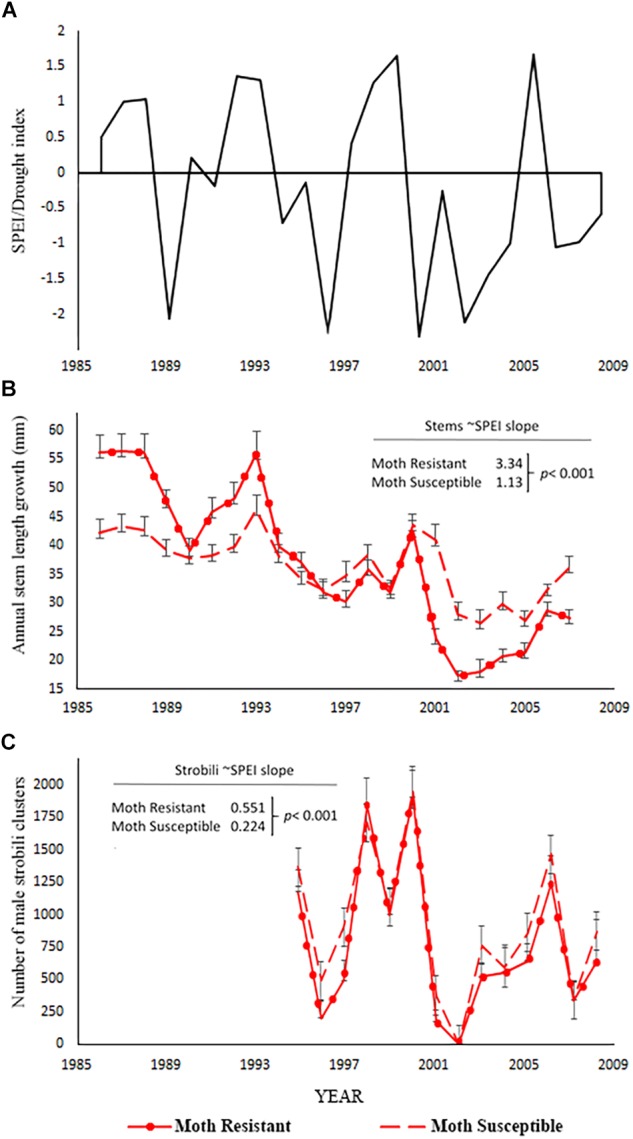
Data for the moth resistant and susceptible tree comparisons (H2). **(A)** Standardized precipitation-evapotranspiration index, SPEI from the DRI website for the 12 months preceding the completion of annual stem growth (Abatzoglou et al., 2017) for which negative numbers indicate greater drought stress. SPEI for the strobili count analysis is different from the one used for stem measures as it is based on the 12 months prior to pollen production in May. **(B)** Annual stem growth mean ± SE for resistant and susceptible trees. The coefficients provided are the slopes of the stem growth relationship to SPEI from the mixed model analysis in Table 5A and the *p*-value is the test of significant difference between slopes for moth resistant versus moth susceptible trees. **(C)** Number of male strobili clusters mean ± SE for the resistant and susceptible trees. The coefficients provides are the slopes of the male strobili count relationship with SPEI from the mixed-model analysis in Table 5B and the *p*-value is the test of significant difference between slopes for moth resistant versus moth susceptible trees.

We hypothesized that moth susceptible trees suffering chronic herbivory before drought would suffer the greatest declines in performance during drought. However, two lines of evidence show the opposite result or are inconsistent with this hypothesis. First, the stem growth of moth resistant trees was more negatively affected by drought than the stem growth of moth susceptible trees (Figure 3). Moth resistant trees had a significant positive relationship between stem growth and SPEI (slope = 3.34, *p* < 0.001; Table 5A) meaning that stem growth declined with increasing drought stress. The slope of the SPEI regression for moth susceptible trees is much less (slope = 1.13, *p* = 0.0002 for contrast with resistant tree slope, Table 5A). This analysis supports the finding of a smaller decline in stem growth for moth susceptible trees with drought (Figure 3) than moth resistant trees. Moth resistant trees had a significant positive relationship between male strobili counts and SPEI (slope = 0.551, *p* < 0.001; Poisson link function, Table 5B and Figure 3C) meaning that male strobili counts declined with increasing drought stress. Moth susceptible trees, however, had a smaller slope for the relationship with SPEI (slope = 0.225, *p* < 0.001; Poisson link function, Table 5B and Figure 3C), suggesting that male function in susceptible trees was less affected by drought than resistant trees.

**Table 5:** Repeated measures, mixed models were used to test for fixed effects of resistant versus susceptible tree groups, the climate proxy, SPEI, and their interaction on the performance measures of **(A)** stem length growth and **(B)** number of male strobili clusters.

(A) Annual stem length growth: linear model.					
	**Value**	**Std. error**	**DF**	***t*-value**	***p*-value**

Intercept (=resistant)	37.70	1.62	965	23.271	0.0000
Susceptible	-0.79	2.29	48	-0.348	0.7295
SPEI (=resistant slope)	3.34	0.422	965	7.918	0.0000
Susceptible: SPEI	-2.21	0.584	965	-3.791	0.0002

**(B) Number of male strobili clusters: generalized linear model: poisson link function.**					

	**Estimate**	**Std. error**	***z*-value**	**Pr( > |*z*|)**	

Intercept (=resistant)	6.798	0.153	44.56	0.0000	
Susceptible	0.356	0.242	1.47	0.1420	
SPEI (=resistant slope)	0.551	0.002	230.59	0.0000	
Susceptible^∗^SPEI	-0.326	0.004	-75.53	0.0000	


*Coefficients and significance testing for fixed factors for testing H2 are in the table below. The comparisons of interest are primarily the slopes of the climate proxy, SPEI, covariate and how it varies across resistant and susceptible tree categories. Both models include individual trees as random factors. The intercept represents resistant trees and SPEI is the slope with SPEI for resistant trees. Susceptible and susceptible^∗^SPEI are the degree to which the main effect and slope differ from the resistant trees (test of H2).*

### Hypothesis 3: Moth Killed Stems on Susceptible and Resistant Trees Within Area One

**FIGURE 4:**
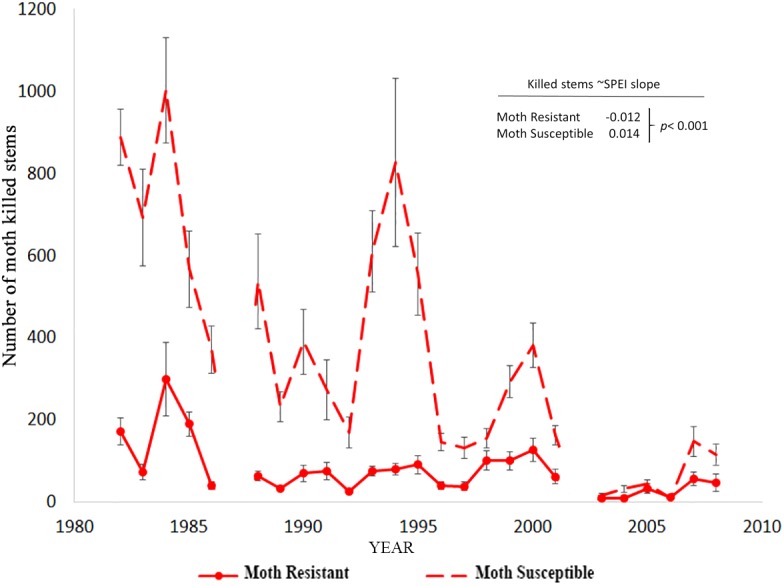
The decline in moth killed stem counts on large susceptible trees through time in comparison to resistant trees. There is a significant positive relationship between moth attack and the climate proxy, SPEI, for susceptible trees (Table 6).

We hypothesized that the number of moth killed stems on trees would increase with drought-induced stress. However, in contrast with the hypothesis, moth herbivory declined substantially with drought for moth susceptible trees as indicated by the significant positive relationship slope with SPEI (slope = 0.014, *p* < 0.001; Poisson link function, Table 6). The decline through time as SPEI decreases is seen in the moth killed stem count data for susceptible trees (Figure 4). According to our model, resistant trees saw a slight increase in herbivory with increasing drought (slope = -0.012, *p* < 0.001; Poisson link function, Table 6).

**Table 6:** The relationship between moth killed stems model as a Poisson response variable and the climate proxy, SPEI, for large resistant and susceptible trees (as assessed at the beginning of monitoring) was tested with a repeated measures, mixed model analysis with SPEI as a covariate (H3).

	Estimate	Std. error	*z*-value	Pr( > |*z*|)
Intercept (=resistant)	6.102	0.028	217.08	0.0000
Susceptible	0.073	0.040	1.86	0.0636
SPEI (=resistant slope)	-0.012	0.002	-6.06	0.0000
Susceptible^∗^SPEI	0.026	0.003	9.29	0.0000


*Individual trees were modeled as random factors in time. The intercept represents the resistant category of trees and the slope coefficient for SPEI represent the relationship between moth killed stems and SPEI for resistant trees. The coefficient for susceptible trees and susceptible^∗^SPEI represent the degree to which susceptible trees differ from resistant trees in intercept and slope, respectively (Figure 4).*

## Discussion

### The Importance of Long-Term Data to Detect Differential Responses

Long-term data can yield unexpected results (Jackson et al., 2018), non-linear or non-additive responses (Rudgers et al., 2018), and distinct answers from predictions based on spatial variation (Estiarte et al., 2016). In long-term studies up to 32 years in duration, we found variation in the response to climate change as measured by stem growth, mortality, and male strobili production across spatial variation in soil type and between herbivore susceptible and resistant trees interspersed on a fine spatial scale. A landscape level study of *P. edulis* showing greater mortality in coarse-textured soils led us to hypothesize that the coarse, cinder soil sites where pinyon grew more slowly before drought (Cobb et al., 2002) should also suffer greater declines in performance during drought (Peterman et al., 2013), however, we found the opposite. Similarly, poor performance of herbivore susceptible trees before drought and positive responses of the herbivore to tree stress led us to hypothesize greater effects of drought on susceptible trees, however, we again found the opposite. Instead moth attack rates declined and susceptible trees showed very little effect of drought on tree performance. These changes in tree performance and impacts of herbivory across years would not have been detected without long-term data collection that spanned drought and non-drought years. Because of the cumulative multi-year effects of drought on plant growth, a single drought year or even a decade of study in a more constant environment does not reflect the shifts in fundamental patterns that are revealed with changing environmental conditions over many years.

### Spatial Variation in Relative Performance Through Time

Before a series of drought years, most indicators suggested that trees growing in the cinder soil areas were under higher abiotic stress as measured by water availability, soil nutrients, plant growth, reproduction, conelet mortality, and insect attack (Christensen and Whitham, 1991; Gehring and Whitham, 1994; Cobb et al., 1997; Swaty et al., 1998; Looney et al., 2012). Based on these findings, we hypothesized (H1) that *P. edulis* growing in the coarse soils (area one) would suffer greater declines with drought in fitness-related traits including survival, stem growth, and male strobili production. This hypothesis was not supported by the data. The cinder soil area (area one), had equal or lesser mortality than other areas and had minor declines in growth and male strobili production with increasing drought stress through time relative to areas two and three. We suggest two explanations for these observations, which may act together and support the hypothesis that most tree species are relatively close to their hydraulic limits (Choat et al., 2012).

First, pinyons in area one that survive in the stressful water and nutrient poor cinder soil environment are adapted and/or acclimated to low water conditions. The relatively strong performance of trees in the cinder soil area through severe and repeated drought years suggests that they may have previously adapted and/or acclimated to chronic water stress due to the low water holding capacity of cinder soils. There may have been strong natural selection since trees established in this area in the 1000 years since the volcanic eruptions that established these cinder fields. Development of root and hydraulic characteristics via acclimation to dry soils may lead to trees with improved acclimation to low water conditions that occur during drought (Brunner et al., 2015; Lübbe et al., 2016). For example, *P. edulis* seedlings grown in cinder soils from area one had 1.5-fold more root biomass and 47% higher ectomycorrhizal fungal colonization than seedlings grown in sandy-loam soils from area two (Gehring and Whitham, 1994). Greater investment in roots and ectomycorrhizal fungi could be beneficial during drought when soil resources are further limited. In support of this hypothesis, nitrogen fertilization of *P. edulis* reduced investment belowground and contributed to high mortality during drought in New Mexico (Allen et al., 2010). Greater investment in roots has been associated with resistance to extreme drought in other pines (Matías et al., 2014; Kolb et al., 2016b), but is not always associated with greater drought survival (Kolb et al., 2016b). Thus, acclimation and adaptation of pinyons to chronically stressful cinder soils may have resulted in their higher performance during current droughts than pinyons growing on nearby soils that were less stressful under non-drought conditions. Studies on other plant species suggest that both adaptation and within and across generation acclimation via plasticity are likely to contribute to the drought tolerance of cinder areas trees (Khalil and Grace, 1992; Franks et al., 2007; Aitken et al., 2008; Yakovlev et al., 2012; Latzel, 2015; Trujillo-Moya et al., 2018). A definitive test of the importance of acclimation and adaptation to these patterns in *P. edulis* requires common garden or greenhouse experiments.

Second, abiotic and biotic differences among areas also may contribute to differential performance under drought. In particular, smaller amounts of precipitation in drought years may be more available in coarse cinder soils where infiltration is rapid (Looney et al., 2012; Peterman et al., 2013; Kolb, 2015). Supplemental watering during drought had a larger positive impact on *P. edulis* growing in young, coarse volcanic soil than in older soils derived from the same parent material (Looney et al., 2012). These data, and ours, are consistent with the “inverse texture hypothesis” of Noy-Meir (1973) which proposes that plants growing in coarse-textured soil experience less water stress than plants growing in fine-textured soil because precipitation is more readily lost from the surface of fine-textured soils via evaporation and/or runoff. Sala et al. (1989) suggested a cut-off for reversal of the soil texture effect at 370 mm of precipitation per year that suggests the potential for year-to-year changes in the effects of soil texture on performance. Interactions with soil microbes also vary with soils, drought, and stress in *P. edulis* (Gehring and Whitham, 1994; Gehring et al., 1998, 2014; Swaty et al., 1998; Gordon and Gehring, 2011) and can have important effects on tree performance (Gehring et al., 2014, 2017). Ectomycorrhizal fungi of the genus *Geopora* promoted drought tolerance in *P. edulis* (Gehring et al., 2014, 2017) and dominated on cinder soils both prior to and during drought (Gehring et al., 1998; Gordon and Gehring, 2011), but were uncommon in non-cinder soils, including area two (Gehring et al., 1998). Thus, variation across the landscape in mycorrhizal symbiont interactions could contribute to variation in drought tolerance, alone or in conjunction with pinyon genetic variation and soil moisture properties.

Our findings that male reproduction was negatively affected by drought supports other research documenting the potential sensitivity of pinyon pine reproduction to climate change. Cone production has declined broadly across the landscape (Redmond et al., 2012) and pollen germination is impaired by the temperatures that *P. edulis* is predicted to experience as climate changes (Flores-Renteria et al., 2018). In this long-term study, we also saw declines in the production of male strobili with drought. While sporadic reproduction and repeated measures on trees experiencing mortality obscure these trends in the raw data (Figure 1C) the analysis in Table 3B supports male strobili declines with drought. Areas one and two show differential responses in strobili production to drought with area one responding less to drought stress and area two responding more (Figure 1C and Table 3B). Strobili production was of intermediate responsiveness to drought at area three which means strobili did not follow exactly the same pattern as stem growth. Area three has older, volcanic substrate soil that is relatively low in phosphorus (Selmants and Hart, 2010). Because lower phosphorus has been associated with lower allocation to male reproduction (Lau and Stephenson, 1994; Fujita et al., 2014) this might be an area to pursue in future studies of pinyon allocation to growth versus male reproduction.

### Decline in Moth Resistant Tree Performance and Moth Attack During Drought

Previous work on the stem-boring moth and its interactions with pinyon pine has shown that this herbivore decreased trunk and branch growth of pinyon pine by ∼45% and shifted reproductive allocation away from female cones and toward male strobili (Whitham and Mopper, 1985; Cobb et al., 2002; Mueller et al., 2005b). This led to the hypothesis that susceptible trees would not fare well with the added stress of drought (H2). Other evidence, such as poor survival of moth resistant trees relative to susceptible trees during drought (Sthultz et al., 2009a) and poor performance of the offspring of moth resistant trees relative to susceptible trees in a common garden (Gehring et al., 2017), has been accumulating since the start of the monitoring program described here that contradicts H2. Our long-term observations of tree growth through time also indicates that moth resistant trees are less tolerant of long-term drought than moth susceptible trees suggesting tradeoffs in which moth resistant trees perform best under normal conditions, but susceptible trees perform best during drought conditions.

Intraspecific variation in drought tolerance has been observed within populations of other species of pine (Gaspar et al., 2013; Kolb et al., 2016b). In ponderosa pine (*P. ponderosa)*, most of the variation in drought-adaptive traits of southern populations occurred within populations (Kolb et al., 2016b). While there is abundant evidence that drought tolerance varies among conifer populations (Moran et al., 2017), the variation among moth resistant and susceptible trees within area one suggests that within population variation is also significant and important to evaluate for reforestation programs where warmer, drier climates are projected.

Our results from male strobili counts demonstrate greater declines under drought for moth resistant trees. In a study conducted before the 2002 mortality event, Cobb et al. (2002) showed that greater strobili production on moth susceptible trees resulted in 1.5-fold greater pollen production from moth susceptible than moth resistant trees. Our data indicate that drought is likely to increase this difference; declining strobili production following drought suggest that resistant trees are even less likely to serve as pollen donors under drought. Research on pollen viability conducted on cinder soils showed that *P. edulis* pollen was highly sensitive to the temperatures predicted with climate change during both the dispersal and germination stages of development (Flores-Renteria et al., 2018). Further research is necessary to determine if resistant and susceptible trees now differ in pollen viability, potentially further shifting male function to moth susceptible, drought tolerant trees. We acknowledge that female cone production, which was not described in this study, may be a more important limitation to reproduction. Female cone production has declined precipitously in the southwest with drought (Redmond et al., 2012).

The decline in moth attack rates with drought was unexpected because earlier studies found that trees growing under favorable, non-drought conditions, moth resistant trees produced two–three times more wound resin than susceptible trees, which is important in resisting damage from stem-boring moths (Mopper et al., 1991; Cobb et al., 1997). Hence, we expected that the moth would continue to increase in numbers on trees further stressed by drought (H3), consistent with the plant stress hypothesis (White, 1969, 1976). Instead, we found that with further increased drought stress, moth attack rates declined. One interpretation of this decline would be that there is a direct impact of drought conditions on moth overwintering, survival, and growth. The alternate interpretation would be an intermediate stress hypothesis in which low and high stress trees are not suitable as hosts for stem-borers because of a tradeoff in which low stress results in vigorous growth with too much resin production that kills larvae, while high stress results in little resin, but too little growth to accommodate tunneling stem-borers. In this scenario, only intermediate stress results in high enough stem growth for stem-borers to thrive and a reduction in resin production to a level that moth larvae can tolerate. This second hypothesis is supported, in part, by experimental water and fertilizer treatments in which experimentally reduced-stress trees suffered significantly less moth herbivory than control trees (Cobb et al., 1997).

There are complex interactions between plant growth and herbivory in this system. Resistant trees grow more slowly than susceptible trees before insect attack begins on mature trees as demonstrated by tree ring studies (Ruel and Whitham, 2002) and common garden studies on maternal families (Gehring et al., 2017). Once herbivory begins, susceptible trees experience lower growth rate and low cone production. This study, and other accumulating evidence, suggests that the relatively good performance of insect susceptible trees during drought is due to a combination of drought resistance and release from herbivore pressure. Thus, periodic drought may help maintain susceptible trees in this system. While cone and seed production is very low during drought, it is likely to shift toward favoring susceptible trees which contribute more pollen during dry times, as well as potentially producing more female cones due to reduced losses of cone-bearing shoots to moth herbivory.

## Author Contributions

AW contributed to data collection, data analysis, and led the writing of the manuscript. NC contributed to the design of the work and the collection of data. LF-R contributed to analysis and figures and revised the manuscript. CG helped design the study, collect the data, and write the manuscript. SM helped collect the data and revise the manuscript. TW helped design the study, collect the data, and revise the manuscript.

## Conflict of Interest Statement

The authors declare that the research was conducted in the absence of any commercial or financial relationships that could be construed as a potential conflict of interest.
